# Resistin in Dairy Cows: Plasma Concentrations during Early Lactation, Expression and Potential Role in Adipose Tissue

**DOI:** 10.1371/journal.pone.0093198

**Published:** 2014-03-27

**Authors:** Maxime Reverchon, Christelle Ramé, Juliette Cognié, Eric Briant, Sébastien Elis, Daniel Guillaume, Joëlle Dupont

**Affiliations:** 1 INRA, UMR 7247 Physiologie de la Reproduction et des Comportements, Nouzilly, France; 2 CNRS, UMR6175 Physiologie de la Reproduction et des Comportements, Nouzilly, France; 3 Université François Rabelais de Tours, Tours, France; 4 Institut Français du Cheval, Nouzilly, France; Duke University Medical Center, United States of America

## Abstract

Resistin is an adipokine that has been implicated in energy metabolism regulation in rodents but has been little studied in dairy cows. We determined plasma resistin concentrations in early lactation in dairy cows and investigated the levels of resistin mRNA and protein in adipose tissue and the phosphorylation of several components of insulin signaling pathways one week post partum (1 WPP) and at five months of gestation (5 MG). We detected resistin in mature bovine adipocytes and investigated the effect of recombinant bovine resistin on lipolysis in bovine adipose tissue explants. ELISA showed that plasma resistin concentration was low before calving, subsequently increasing and reaching a peak at 1 WPP, decreasing steadily thereafter to reach pre-calving levels at 6 WPP. Plasma resistin concentration was significantly positively correlated with plasma non esterified fatty acid (NEFA) levels and negatively with milk yield, dry matter intake and energy balance between WPP1 to WPP22. We showed, by quantitative RT-PCR and western blotting, that resistin mRNA and protein levels in adipose tissue were higher at WPP1 than at 5 MG. The level of phosphorylation of several early and downstream insulin signaling components (IRβ, IRS-1, IRS-2, Akt, MAPK ERK1/2, P70S6K and S6) in adipose tissue was also lower at 1 WPP than at 5 MG. Finally, we showed that recombinant bovine resistin increased the release of glycerol and mRNA levels for ATGL (adipose triglyceride lipase) and HSL (hormone-sensitive lipase) in adipose tissue explants. Overall, resistin levels were high in the plasma and adipose tissue and were positively correlated with NEFA levels after calving. Resistin is expressed in bovine mature adipocytes and promotes lipid mobilization in adipose explants *in vitro*.

## Introduction

In the dairy cow, late gestation and early lactation are periods marked by major changes in the sensitivity and responses of tissues to hormones involved in homeostasis, such as insulin [1]. Indeed, during these periods, there is a moderate decrease in peripheral tissue insulin sensitivity, promoting the mobilization of non esterified fatty acids (NEFAs) and amino acids and facilitating the preferential use of nutrients by the fetus or mammary gland [2]. The decrease in insulin sensitivity occurring in adipocytes during late gestation and early lactation in dairy cows remains poorly understood. Insulin acts by binding to the insulin receptor (IR), a tyrosine kinase receptor, on cells. Following insulin binding, the IR phosphorylates various substrates, including IRS-1 and IRS-2, which interact with several intracellular proteins to activate different signaling pathways, including the PI3K/Akt and MAPK ERK1/2 pathways [3]. Sadri *et al*. studied the expression of genes encoding components of the insulin receptor signaling pathway in adipose tissue during the dry period and in early lactation, in dairy cows [4]. They observed a significant decrease in insulin-responsive glucose transporter (GLUT4) gene expression in subcutaneous adipose tissue around the time of parturition. However, the levels of phosphorylation of IR signaling components have never been investigated. Adipokines — factors secreted by the adipose tissue — may be involved. In particular, resistin is known to decrease insulin sensitivity in rodents, whereas its effect in humans is unclear [5]. Resistin is a protein consisting of 108 amino acids in humans, 114 amino acids in mice, and 109 amino acids in cattle; it belongs to the "resistin-like molecules" or "FIZZ" (found in inflammatory zone) family [6]. It consists of homodimers linked by disulfide bridges. Resistin is produced directly by the adipocytes in mice, whereas it is produced by macrophages and transported to adipocytes in humans [7], [8]. Plasma resistin levels are correlated with the degree of insulin resistance in mice, whereas conflicting results have been reported concerning this aspect in humans [6]. In bovine species, the localization in adipose tissue and the role of resistin in lipolysis are still unknown.

In mice, plasma and adipose tissue levels of resistin decrease in response to thiazolidinediones (insulin sensitizers) and increase during obesity [9]. Very little is currently known about the mode of action of resistin. No receptor has yet been clearly identified and the signaling pathways used remain unclear. Recent studies have suggested that resistin may bind a receptor tyrosine kinase called ROR1 (receptor tyrosine kinase-like orphan receptor) in murine pre-3T3-L1 adipocytes [10], or to TLR4 (Toll-like receptor 4) in the hypothalamus of mice [11]. The adipose tissue of dairy cows also produces several adipokines [4], [12], [13], [14], including resistin [15]. Komatsu *et al*. showed that levels of resistin gene expression in the adipose tissue were significantly higher in lactating than in non lactating cows, whereas the opposite pattern was observed in the mammary gland [15]. However, plasma resistin concentration has never been determined during lactation in the dairy cow and the role of resistin in bovine adipose tissue has never been studied.

We investigated the profile of plasma resistin, insulin, glucose and non esterified fatty acid (NEFA) concentrations around the time of parturition and at the start of the first lactation in dairy cows. For the second lactation in the same animals, we then investigated mRNA and protein levels for resistin and the phosphorylation rates of several insulin receptor signaling components *in vivo* in subcutaneous adipose tissue in early lactation and mid-gestation. Finally, for the fifth lactation in the same animals, we analyzed the effects of bovine recombinant resistin on lipolysis *in vitro* in adipose tissue explants performed between one and two months after calving.

## Materials and Methods

### Animals

All experimental protocols were approved by an ethics committee (“Comité d’Ethique en Expérimentation Animale Val de Loire” CEEA VdL, protocol registered as n°2012-09-6), and were carried out in accordance with the guidelines of the French Council for Animal Care.

#### First experiment

Eight Holstein dairy cows were studied during the first months of their first and second lactations. Dairy cows were managed in loose housing conditions throughout the study. Cows were fed *ad libitum*, throughout the first four months of lactation after calving, with a complete mixed diet composed of 64.5% maize silage, 10% soybean, 15% concentrate, 10% dehydrated alfalfa and 0.5% calcium oxide (CaO). From the fifth month of lactation, the diet consisted of 74.64% maize silage, 10% soybean, 8% concentrate, 7% dehydrated alfalfa, 0.16% CaO and 0.2% mineral and vitamin mixture. Cows were artificially inseminated from seven weeks postpartum (7 WPP), half a day after the detection of estrus.

#### Second experiment

The same dairy cows as for the first experiment were studied during their second lactation. Biopsies of adipose tissue were performed at 1 week postpartum (WPP1) and 5 months of gestation (5 MG), as described below in the section dealing with sample collection.

#### Third experiment

Subcutaneous adipose tissue for adipose tissue explants was collected from the same dairy cows as for the first and second experiment (n = 8) between one and two months after calving during their fifth lactation. Animals were slaughtered at a local abattoir (INRA, PRC Unit, Nouzilly).

### Body weight, milk yield, feeding and Energy Balance

After each milking, cows were automatically weighted (software RIC version RW1.7). Only the morning live body weight was used for weight analyses because the afternoon body weight was more variable. All cows were milked twice daily. At the entrance of the milking parlour, the cows were identified by an electronic collar and milk yield of each cow was automatically recorded (software Manufeed 500 pro, vc5 version 2.011.14).

Primiparous cows were fed *ad libitum* with two total mixed rations according to their stage of lactation using the INRA French feeding system [16] as described in [16]. Dry matter intake was determined from the intake of fresh matter and the dry matter content of each feed of the ration. The chemical composition of each feed is the same as described in [16]. The feeding values of the different feeds were calculated using chemical composition according the methods defined in INRA feeding systems [16]. Energy balance corresponds to the difference between energy needs for body maintenance, pregnancy and lactation, and energy intake.

### Sample collection

Blood samples were collected from the tail vein immediately before food distribution, once per week (from 4 weeks before calving until 22 weeks after calving). They were centrifuged at 3000×g for 10 min at 4°C and plasma was stored at −20°C until its use for assays.

During the second lactation, adipose tissue biopsies were carried out on the same animals at 1 week post partum (WPP 1) and 5 months of gestation (5 MG; at this stage of gestation, the animals were still lactating). Cows were fasted for 12 hours before surgery and anesthesia was induced by intravenous (IV) injections of 12 to 14 mg of xylazine (Rompun, Bayer, Leverkusen, Germany) and subcutaneous (SC) injections of 20 mg lidocaine (Lurocaïne, Vetoquinol, Lure, France). Subcutaneous fat was collected from the dewlap under the neck, immediately frozen in liquid nitrogen and stored at −80°C until use. Blood samples were collected on the day of the biopsy.

### Plasma metabolites and insulin assays

Non esterified fatty acids (NEFA) and glucose were determined by enzymatic colorimetry, on a multiparameter analyzer (KONE Instruments Corporation, Espoo, Finland). Plasma insulin was determined by RIA, as previously described [17].

### Resistin ELISA assay

Plasma bovine resistin levels were determined with a commercially available bovine resistin enzyme-linked immunosorbent assay (ELISA) (reference: E90847Bo (96 tests), distributed by Euromedex, France; supplier: USCNLife), according to manufacturer’s protocol, with an intra-assay coefficient of variation < 6%.

### Immunohistochemistry

Adipose tissue samples from the left side of the carcass were fixed by incubation with Bouin’s solution for 24 h at room temperature, dehydrated, embedded in paraffin, and cut into 5 μm-thick sections. The paraffin was then removed from the sections, which were hydrated and microwaved in antigen-unmasking solution for 5 minutes (Eurobio, Les Ulis, France) and then allowed to cool to room temperature. Sections were then washed in PBS for 5 minutes and immersed in peroxidase-blocking reagent for 10 minutes at room temperature, to quench endogenous peroxidase activity (DAKO Cytomation, Dako, Ely, UK). Adipose tissue sections were incubated for 20 min in PBS supplemented with 5% lamb serum, to eliminate nonspecific background. They were then washed in a PBS bath for five minutes and incubated overnight at 4°C with PBS supplemented with a rabbit primary antibody raised against human resistin (ab14323) from Abcam. According to the manufacturer, this antibody should recognize bovine resistin. Sections were washed twice, for 5 minutes each, in a PBS bath, and were then incubated for 30 minutes at room temperature with a “ready-to-use” polymer-HRP-conjugated anti-rabbit antibody (DakoCytomation Envision Plus HRP System, Dako, Ely, UK). Finally, sections were washed twice in PBS and staining was detected by incubation at room temperature with 3,3’-diaminobenzidine tetrahydrochloride (Liquid DAB+Substrate Chromogen System, DakoCytomation). We used primary antibodies against rabbit IgG as negative controls.

### Total RNA extraction

Total RNA was extracted from 250 mg of dewlap subcutaneous adipose tissue on ice, with an Ultraturax homogenizer and 8 ml of QIAzol lysis reagent (Qiagen, Courtaboeuf, France). Chloroform (1.6 ml) was added to each sample. Tubes were shaken for 15 seconds and left at room temperature for 5 minutes before centrifugation (5000×*g*, 15 minutes, 4°C). The aqueous phase was mixed with an equal volume of ethanol 70% (v:v) and total RNA was purified with an RNeasy Midi Kit (Qiagen, France), according to the manufacturer’s protocol. An RNase-free DNaseI (Qiagen) treatment was performed during the purification process. The RNA was eluted in RNase-free water, and the solvent was allowed to evaporate off, without heating, for 1.5 hours in a Thermo Savant SPD1010 SpeedVac System. The RNA was then stored at −80°C until use. The amount of RNA was determined with a NanoDrop Spectrophotometer (Nyxor Biotech, Paris, France) and RNA quality was assessed with an Agilent 2100 Bioanalyzer, using an RNA 6000 Nano assay protocol (Agilent Technologies, Massy, France). The RNA integrity number (RIN) for each RNA sample is shown in the Table S1.

### Real-time quantitative RT-PCR (RT-qPCR)

Reverse transcription was performed as previously described [18]. Resistin cDNAs were quantified by real-time PCR with SYBR Green Supermix (Bio-Rad, Marnes la Coquette, France) and 250 nM specific primers (Invitrogen™ by Life Technologies™, Table 1), in a total volume of 20 μl, in a MyiQ Cycle device (Bio-Rad). Samples were tested in duplicate on the same plate, and PCR amplification with water, instead of cDNA, was performed systematically as a negative control. After incubation for 2 minutes at 50°C and denaturation for 10 minutes at 95°C, samples were subjected to 40 cycles of 30 seconds at 95°C, 30 seconds at 60°C and 30 seconds at 72°C, and the melting curve was determined. Primer efficiency (E), determined for the bovine resistin, ATGL and HSL primers on serial dilutions of a pool of the cDNAs obtained, was 1.90, 1.85 and 2.0, respectively. The geometric mean of four reference genes (PPIA, RPL19, ACTB and GAPDH) was used to normalize gene expression. The relative amounts of gene transcripts (R) were calculated according to the equation R  =  (Egene−Ct gene)/[geometric mean (EPPIA−CtPPIA; ERPL19−CtRPL19; EACTB−CtACTB; EGAPDH−CtGAPDH)], where Ct is the cycle threshold and E the PCR efficiency for each primer pair

**Table 1:** Oligonucleotide primers sequences.

Abbrev.	name	gene ID	forward 5'-3'	reverse 5'-3'	Size bp	efficiency
PPIA	Cyclophilin A	NM_178320	GCATACAGGTCCTGGCATCT	TGTCCACAGTCAGCAATGGT	217	2.01
RETN	Resistin	NM_183362	AGTCCACAGAGAGGCACCTG	TGGTGACCTCCTGGATCTTC	133	2.04
RPL19	Ribosomal protein L19	BC102223	AATCGCCAATGCCAACTC	CCCTTTCGCTTACCTATACC	156	2.20
ACT	Beta Actin	D12816	ACGGAACCACAGTTTATCATC	GTCCCAGTCTTCAACTATACC	180	2.05
ATGL	Adipose Triglyceride Lipase	FJ798978	AAGCTGGTGCCAACATCATC	TAGCAATCAGCAGGCAGAAT	130	2.01
HSL	Hormone-Sensitive Lipase	NM_00108	GAGACTGGCATCAGTGTGAC	TTGCTAGAGACGATAGCACCT	199	1.98
CD68	CD68	NM_001045902	GAGGCAATAGGAGACTACAC	TGAATCCGAAGCTGAGCTGT	220	2.01
GAPDH	Glyceraldehyde 3 phosphate dehydrogenase	NM_001034034	TTCAACGGCACAGTCAAGG	ACATACTCAGCACCAGCATCAC	119	2.18

### Recombinant proteins and antibodies

Recombinant bovine resistin was produced by Cliniscience (Nanterre, France); the reference number of the product used was: Recombinant Bovine Resistin-E Coli-CSB-EP019573BO. Recombinant human insulin used for culture treatment was obtained from Sigma (St Louis, MO, USA). Rabbit polyclonal antibodies against phospho-ERK1/2 (Thr202/Tyr204), phospho-p38 (Thr180/Tyr182), phospho-Akt (Ser 473), Akt, phospho-P70S6 kinase (Ser424/Thr421), phospho-S6 (Ser235/236), P70S6, S6 and phospho-AMPK alpha Thr172 were obtained from New England Biolabs Inc. (Beverly, MA). Rabbit polyclonal antibodies against AMPKalpha, IRS-1 and IRS-2 were purchased from Upstate Biotechnology Inc. (Lake, Placid, NY, USA). Rabbit polyclonal antibodies against IGF-1R beta subunit (C20), IR beta subunit (C19), ERK2 (C14) and p38 (C20) were purchased from Santa Cruz Biotechnology (Santa Cruz, CA). Mouse monoclonal antibodies against vinculin were purchased from Sigma (St. Louis, MO, USA). PY20 antibodies were obtained from BD Biosciences (Le Pont de Claix, France). Rabbit monoclonal antibodies to human Adiponectin (C45B10) were from Cell Signaling Technology (Ozyme, Saint Quentin Yvelines, France). Rabbit polyclonal antibodies against human resistin were obtained from Abcam (reference: ab14323). On immunoblots, this antibody recognized a band at about 12.5 kDa in bovine adipose tissue (Figure S1). It also recognized the recombinant bovine resistin, which migrated at the same molecular weight. We therefore conclude that the band detected in the bovine adipose tissue is probably bovine resistin. All antibodies were diluted 1/1000 for western blotting.

### Isolation of bovine stromal vascular cells and mature adipocytes

Adipose tissue samples were collected from the left side of the carcass immediately after exsanguination. Incisions were made dorsal to the 12th and 13th rib, and a sample approximately 10 cm^3^ in volume and containing a portion of subcutaneous adipose tissue was obtained. Immediately after collection, the samples were placed in sterile ice-cold PBS and transported to the laboratory. Briefly, subcutaneous adipose tissue was separated from the visible collagenous connective tissue. All excised adipose samples were then cut into approximately 2-mm^3^ cubes. The samples were digested in Dulbecco’s modified Eagle’s medium (DMEM; 5.5 mM glucose; PAA, France) supplemented with 2 mg/ml collagenase (Sigma, France) and 2% BSA. They were incubated, with shaking at 230 rpm, for 45 minutes in a 37°C water bath. The digested cell suspension was then filtered through a sterile nylon mesh with 1000-μm pores into a clean 50 ml centrifuge tube. The unwanted connective tissue was retained on the mesh. The cells passing through the filter were centrifuged at 200×*g* for 10 minutes and the floating mature adipocytes in the uppermost layer were collected and washed twice by centrifugation (200×*g*, 10 min). The final pellet (the stromal vascular cell fraction) and the isolated mature adipocytes were then frozen at −80°C until use.

### Adipose tissue explant culture

Subcutaneous adipose tissue was obtained from the same location as for stromal vascular cell and mature adipocyte isolation from dairy cows (*n* = 8 animals during their fifth lactation (same animals as those used for experiment 1 and 2), n = 2 animals per experiment), from a local abattoir. We placed about 3 g of tissue in sterile sodium chloride solution (0.9%) to remove the excess blood, and connective tissue was removed at the initial sampling stage. The remaining tissue was immediately transferred to a 50 ml tube containing DMEM supplemented with l-glutamine (PAA Laboratories GmbH, Cölbe, Germany) supplemented with 100 μg/ml streptomycin and 50 μg/ml gentamicin, used as the basal medium. The tube was immersed in water at 37°C water in a thermo-flask dewar and transported to the laboratory. We dissected 200 mg of the subcutaneous adipose tissue, cutting it into 10 small pieces in sterile conditions. The tissue samples were incubated in triplicate with 3 ml of basal medium, basal medium with various concentrations of recombinant bovine resistin (1, 10 and 100 ng/ml), in the presence or absence of insulin (10^−8^ M), for 4 h, at 37°C, under an atmosphere containing 5% CO_2_. The tissue explants (200 mg) were then collected, immediately frozen in liquid nitrogen and stored at −80°C.

### *In vitro* lipolysis assay

The rate of lipolysis was determined by monitoring glycerol release from the adipose tissue explants into the incubation medium with a determination kit for free glycerol (Sigma, St. Louis, MO, USA).

### Protein extraction and western blotting

Tissue lysates (adipose tissue) were prepared on ice with an Ultraturax homogenizer in lysis buffer A, consisting of 10 mM Tris (pH 7.4), 150 mM NaCl, 1 mM EDTA, 1 mM EGTA and 0.5% Nonidet P-40 supplemented with various protease inhibitors (2 mM PMSF, 10 mg/ml leupeptin, 10 mg/ml aprotinin) and phosphatase inhibitors (100 mM sodium fluoride, 10 mM sodium pyrophosphate, 2 mM sodium orthovanadate), as previously described [19]. The proteins extracted (80 μg) were denatured, subjected to SDS-PAGE in a 12% polyacrylamide gel, transferred onto nitrocellulose membranes and incubated with specific antibodies, as previously described [20], [21]. Proteins were detected by enhanced chemiluminescence (Western Lightning *Plus*-ECL, Perkin Elmer), with a G:Box SynGene (Ozyme) and GeneSnap software (release 7.09.17). The signals detected were quantified with GeneTools software (release 4.01.02). The results are expressed as the intensity signal after normalization, in arbitrary units, as indicated in the figure legends.

### Immunoprecipitation

After normalization for the protein concentration (500 μg) of cell lysates, IR, IRS-1, IRS-2 and IGF-1R were immunoprecipitated from the supernatants with 5 μg of the appropriate antibodies, by incubation overnight at 4°C. The immunocomplexes were precipitated by incubation with 40 μl of protein A-agarose for 1 h at 4°C. The pellets were washed twice with a 1 in 2 dilution of buffer A and then boiled for 4 min in reducing Laemmli buffer containing 80 mm dithiothreitol. Proteins were resolved by SDS-PAGE and transferred to nitrocellulose membranes. Blots were blocked in 2% BSA and probed with the various antibodies, as indicated in the figure legends.

### Statistical analysis

In experiment 1, data for the plasma concentrations of NEFA, glucose, insulin and resistin were analyzed from 4 weeks before calving until 22 weeks post partum (wpp), in a linear mixed model, with the MIXED procedure of SAS software [22] for repeated measurements. For the analysis of these metabolic data, the period studied was divided into six subperiods: period 1: 4 and 2 weeks before calving; period 2: 1 and 2 WPP; period 3: 4 and 6 WPP; period 4: 8 and 10 WPP; period 5: 12 and 14 WPP; period 6: between 16 and 22 WPP. The model included period and animal as fixed effects and the interactions between period and animal. If significant effects were detected, the lsmeans per subperiod were compared, considering *p*<0.05 to be significant. The relationships between quantitative parameters (NEFA, glucose, insulin and resistin concentrations) were investigated by Pearson’s correlation analyses, with the CORR procedure of SAS software [22].

For experiments 2 and 3, all the other experimental data (mRNA and protein levels, and phosphorylation levels) are presented as means ± SEM. One-way analysis of variance (ANOVA) was used to assess the significance of differences (Statview version 5.0, SAS Institute, Inc.). If ANOVA revealed significant effects, the means were compared in Fisher’s test, considering *p*<0.05 to be significant. Different letters indicate significant differences.

## Results

### Plasma resistin levels during the first and second lactation

**Figure 1:**
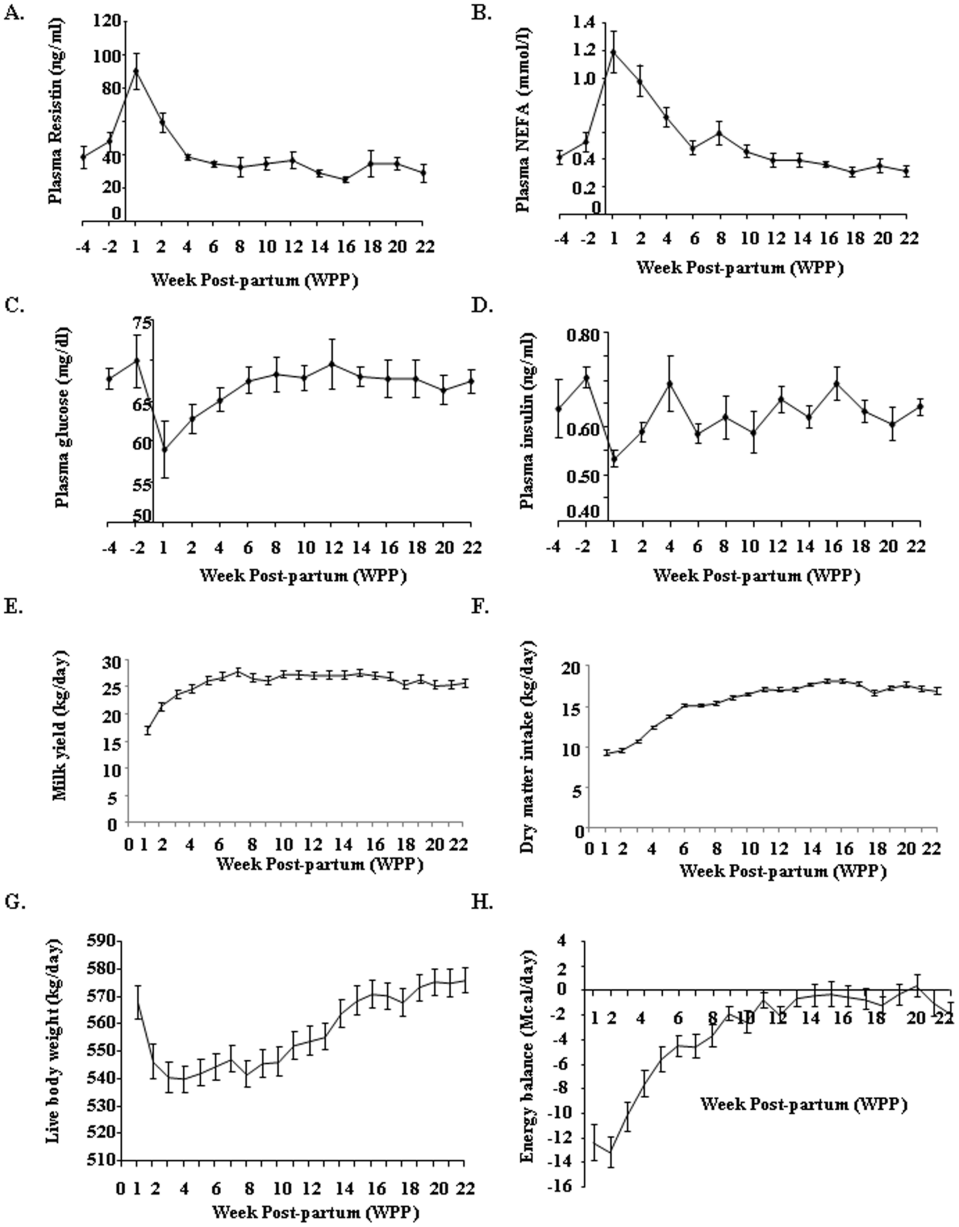
Plasma resistin, NEFA, glucose and insulin levels and zootechnical parameters during the 1st lactation. Changes in plasma (A) resistin, (B) NEFA, (C) glucose, and insulin (D) concentrations in dairy cows, from 1 month before calving until 22 weeks after calving (*n* = 8 animals). Evolution of (E) milk yield, (F) dry matter intake, (G) live body weight and (H) energy balance between week post-partum (WPP) 1 and WPP22. Results are presented as means ± SEM.

In the first experiment, we determined the plasma concentrations of resistin, NEFAs, glucose and insulin in eight Holstein dairy cows, from four weeks before calving to 22 weeks post partum, during their first lactation (figure 1). These concentrations differed significantly before and after lactation (*p*<0.001, Table 2). As expected, plasma glucose and insulin concentrations were lower, whereas plasma NEFA concentration was higher one week after calving than before calving (*p*<0.05, figure 1B to D). Plasma resistin concentrations were low before calving (about 40 ng/ml), subsequently increasing and reaching a peak one week after calving (about 90 ng/ml), decreasing steadily thereafter to reach pre-calving levels at 6 weeks post-partum (Figure 1A, Table 2). Plasma resistin concentration did not change between 6 and 22 weeks post partum (Table 2). A significant correlation between plasma resistin and NEFA concentrations (*r* = 0.43, *p*<0.0001) was observed between four weeks before calving and 22 weeks post partum (Table 3). By contrast, there was no significant correlation between plasma resistin and glucose or insulin concentrations during this period (Table 3).

**Table 2:** Statistical analysis of resistin, NEFA, glucose and insulin plasma levels.

Item	Animal	Period	Animal*Period	Period Post Partum					
	4 wk before calving to WPP22			4 to 2 wk before calving	WPP1 to WPP2	WPP4 to WPP6	WPP8 to WPP10	WPP12 to WPP14	WPP16 to WPP22
Resistin (ng/ml)									
mean	40.15			43.25	75.10	36.42	33.03	32.76	30.26
*P*	0.0001	0.0001	0.52	B	A	C	C	C	C
NEFA (mmol/l)									
mean	0.54			0.47	1.08	0.60	0.52	0.40	0.33
*P*	0.0004	0.0001	0.0011	B,C	A	B	B	C,D	D
Glucose (mg/dl)									
mean	67.12			68.89	61.12	66.38	68.17	70.15	67.40
*P*	0.0001	0.0001	0.03	A	B	A	A	A	A
Insulin (ng/ml)									
mean	0.63			0.68	0.57	0.65	0.60	0.63	0.65
*P*	0.0025	0.0010	0.27	A	B	C	C	C	C

Arithmetic mean values for each studied period are presented. P values of the effects of animal, period, interaction between animal and period for each studied parameter, and P values of the comparison between lsmeans of each parameter and for each of the six studied periods are described. Different letters indicate significant differences at p<0.05.

**Table 3:** Statistical analysis of the relationships between quantitative parameters (NEFA, glucose, insulin and resistin plasma levels during the period 4 weeks before calving to 22 weeks post partum) using Pearson correlations with the CORR procedure of the SAS software.

	NEFA	Glucose	Resistin	Insulin
**NEFA**	1.00			
				
**Glucose**	−0.36	1.00		
*P*	<0.0001			
**Resistin**	0.43	−0.13	1.00	
*P*	<0.0001	0.21		
**Insulin**	−0.30	0.30	0.05	1.00
*P*	0.004	0.003	0.650	

As shown in figure 1E to H, zootechnical parameters concerning milk production, food intake, body weight and energy balance were analyzed from calving to 22 weeks post partum (wpp) during first lactation. As expected, all those parameters significantly varied during the lactation (wpp effect: p<0.0001, Table 4). A significant correlation between plasma resistin and milk yield (*r* = −0.52, *p*<0.0001), plasma resistin and dry matter intake (r = −0.65, *p*<0.0001) and plasma resistin and energy balance (r = −0.62, *p*<0.0001) was observed between 1 and 22 weeks post partum (Table 5). By contrast, there was no significant correlation between plasma resistin and live body weight (Table 5).

**Table 4:** Statistical analysis of live body weight, milk yield, dry matter intake and energy balance.

Item	Animal	Period	Animal*Period	Period Post Partum				
	WPP1 to WPP20			WPP1 to WPP2	WPP4 to WPP6	WPP8 to WPP10	WPP12 to WPP14	WPP16 to WPP22
Live body weight (kg/day)								
mean	557.30			548.07	543.75	549.20	564.53	572.80
*P*	0.0001	0.0001	0.0001	A	A	A	B	C
Milk yield (kg/day)								
mean	25.96			21.97	27.04	27.23	27.46	26.06
*P*	0.0004	0.0001	0.10	A	B,C	B,C	B	C
Dry matter intake (kg/day)								
mean	15.50			10.36	14.78	16.61	17.76	17.18
*P*	0.0001	0.0001	0.004	A	B	C	D	C,D
Energy balance (Mcal/day)								
mean	−3.43			−10.86	−4.59	−1.08	−0.79	−0.44
*P*	0.0001	0.0001	0.06	A	B	C	C	C

Arithmetic mean values for each studied period are presented. P values of the effects of animal, period, interaction between animal and period for each studied parameter, and P values of the comparison between lsmeans of each parameter and for each of the five studied periods are described. Different letters indicate significant differences at p<0.05.

**Table 5:** Statistical analysis of the relationships between quantitative parameters (resistin plasma levels, milk yield, dry matter intake and energy balance during the period 1 to 20 WPP) using Pearson correlations with the CORR procedure of the SAS software.

	Resistin	Milk Yield	Dry matter intake	Energy Balance	Live Body Weight
Resistin	1.00				
					
Milk Yield	−0.52	1.00			
*P*	<0.0001				
Dry matter intake	−0.65	0.75	1.00		
*P*	<0.0001	<0.0001			
Energy Balance	−0.62	0.27	0.77	1.00	
*P*	<0.0001	0.002	<0.0001		
Live Body Weight	−0.10	0.46	0.49	0.08	1.00
*P*	0.34	<0.0001	<0.0001	0.35	

**Figure 2:**
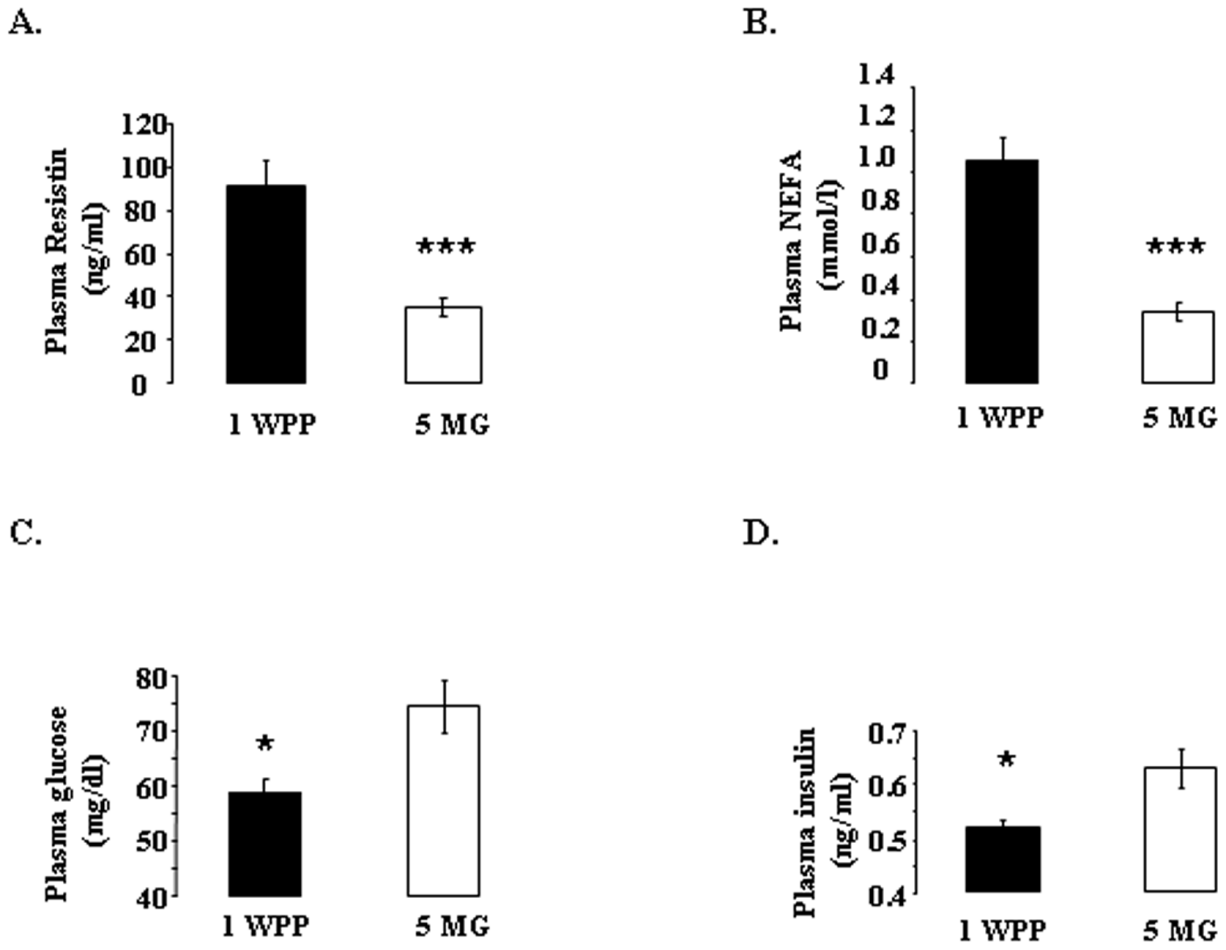
Plasma resistin, NEFA, glucose and insulin levels at 1 week post partum (WPP 1) and 5 months of gestation (5 MG) during the second lactation. Plasma resistin, NEFA, glucose and insulin concentrations measured on the day of adipose tissue biopsy (1 WPP and 5 MG) in Holstein dairy cows. Animals (*n* = 8 in each group) were fasted for 12 hours before surgery Results are presented as means ± SEM. Results are considered significantly different if *p*<0.05. * and *** indicate significant differences at *p*<0.05 and *p*<0.0001, respectively.

In the second experiment, we measured the same plasma variables during the second lactation and, more precisely, on the days on which adipose tissue biopsies were carried out, one week postpartum (1 WPP) and at five months of gestation (5 MG). Plasma resistin and NEFA concentrations were higher (figure 2A and B), whereas plasma glucose and insulin concentrations were lower (Figure 2C and D) one week post partum than at 5 MG. At 1 WPP, fat is being mobilized, whereas at 5 MG, body reserves are being reconstituted.

### Resistin mRNA and protein levels and adiponectin protein levels in the adipose tissue of dairy cows at one week post partum and 5 months of gestation

**Figure 3:**
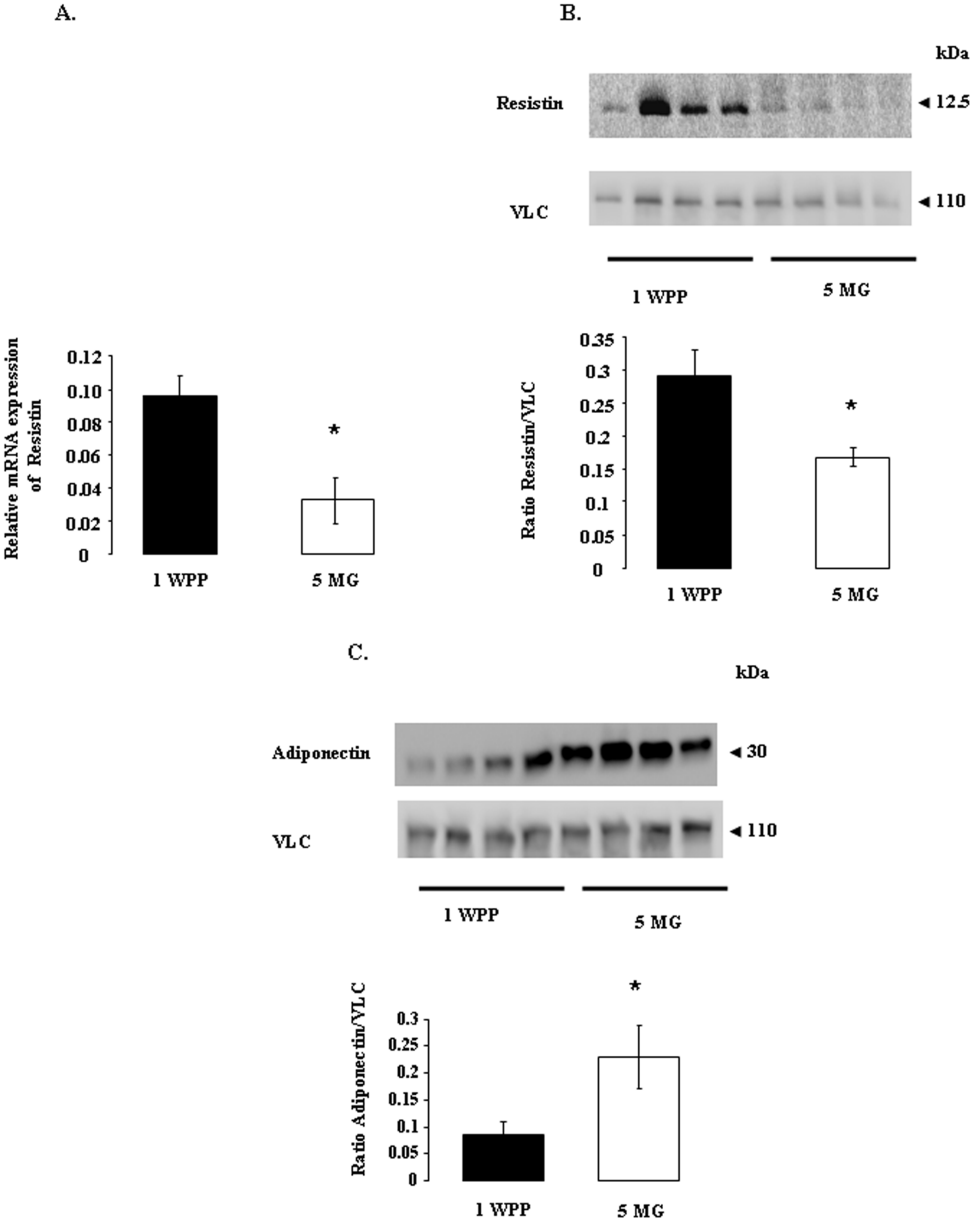
Resistin mRNA and protein levels and adiponectin protein levels in bovine adipose tissue at one week post partum (WPP 1) and 5 months of gestation (5 MG). A. Resistin gene expression was assessed by quantitative reverse transcription-polymerase chain reaction on bovine adipose tissue at one week post partum (WPP 1, *n* = 8 animals) and 5 months of gestation (5 MG, *n* = 8 animals), as described in the materials and methods. Relative expression was measured relatively to the geometric mean of 4 reference gene expression [cyclophilin A (PPIA), GAPDH, Actin B (ACTB), and ribosomal protein L19 (RPL19)] by real-time reverse-transcription PCR. Results are represented as means ± SEM. * indicates a significant difference (*p*<0.05). **B and C**. Resistin (B) and adiponectin (C) protein levels were analyzed by western blotting on bovine adipose tissue at one week post partum (1 WPP, *n* = 8 animals) and 5 months of gestation (5 MG, *n* = 8 animals), as described in the materials and methods. Vinculin (VLC) was used as a loading control. Results are represented as means ± SEM. * indicates a significant difference (*p*<0.05).

We determined resistin mRNA levels in adipose tissue at 1 WPP and 5 MG during the second lactation. Levels of resistin mRNA were higher at 1 WPP than at 5 MG (Figure 3A). We confirmed this result at the protein level by western blotting (figure 3B). At the opposite, we showed that adiponectin protein levels were lower at 1 WPP than at 5 MG (Figure 3C).

### Phosphorylation of IRβ, IRS-1, IRS-2 and IGF-1Rβ in the adipose tissue of dairy cows at one week post partum and 5 months of gestation

**Figure 4:**
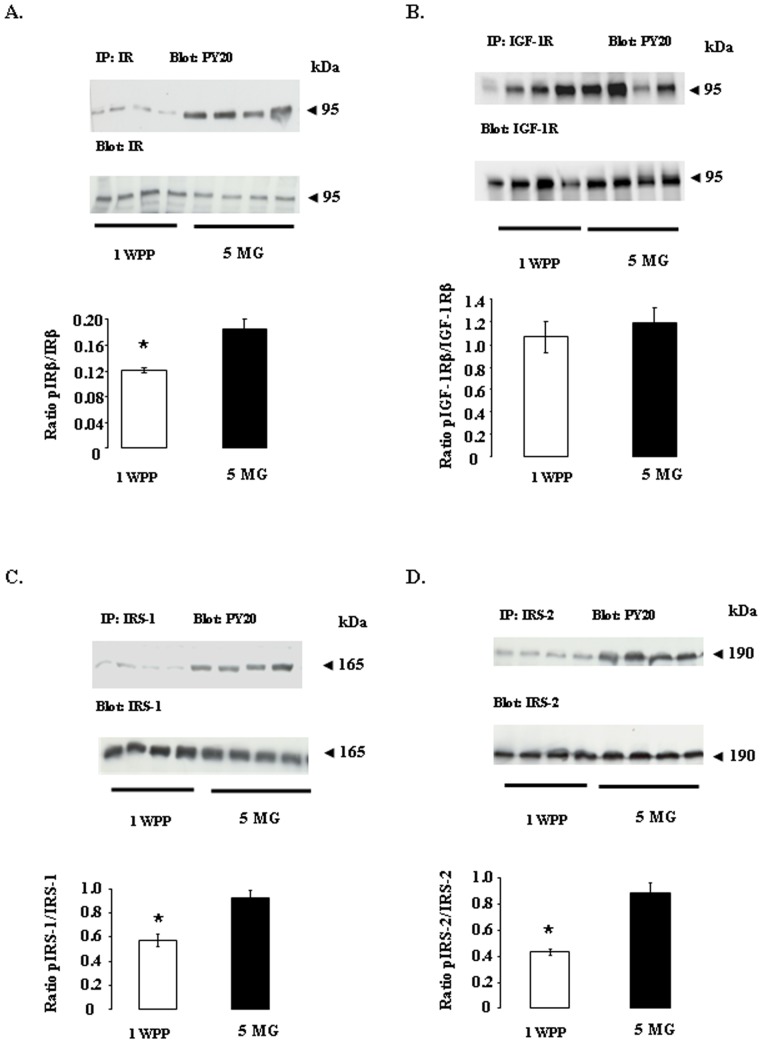
Levels of tyrosine phosphorylation for IR, IGF-1R, IRS-1 and IRS-2 in bovine adipose tissue at one week post partum (1 WPP) and 5 months of gestation (5 MG). Western blots showing the tyrosine phosphorylation levels of IRβ (A), IGF-1Rβ (B), IRS-1 (C) and IRS-2 (D) in adipose tissue lysates from dairy cows at one week post partum (1 WPP, *n* = 8 animals) and 5 of months gestation (5 MG, *n* = 8 animals). Immunoprecipitation (IP) was performed before gel electrophoresis. The antibody used is indicated as follows: IP: molecule X; the immune sera used to determine protein phosphorylation levels are indicated to the left of the gels (e.g., PY20 is directed against anti-phosphotyrosine residues). The levels of phosphorylation of IR, IGF-1R, IRS1 and IRS-2 were normalized with respect to the corresponding total protein, with specific antibodies, as indicated to the left of the gels. The gels show protein bands, which are underlined for each group, with four dairy cows per group. From left to the right, the groups are 1 WPP and 5 MG. Below each gel, the histograms show the mean ± SEM for a total of *n* = 8/group. *indicates a significant difference (*p*<0.05).

Early steps in insulin and IGF-1 receptor signaling (IRβ, IGF-1Rβ, IRS-1 and IRS-2) were first compared in adipose tissue at 1 WPP and 5 MG (Figure 4A to D). Our results suggest that the tyrosine phosphorylation levels of IRβ, IRS-1 and IRS-2 were lower (*P*<0.05) at 1 WPP than at 5 MG (Figure 4A, C and D). By contrast, the IGF-1R beta subunit displayed similar levels of tyrosine phosphorylation at these two stages (Figure 4B). Furthermore, we found that the levels of the IRβ, IGF-1Rβ, IRS-1 and IRS-2 proteins did not differ between 1 WPP and 5 MG (normalized with respect to VLC, data not shown).

### Phosphorylation of MAPK ERK1/2 and p38, Akt, AMPK, P70S6K and S6 in the adipose tissues of dairy cows at one week post partum and 5 months of gestation

**Figure 5:**
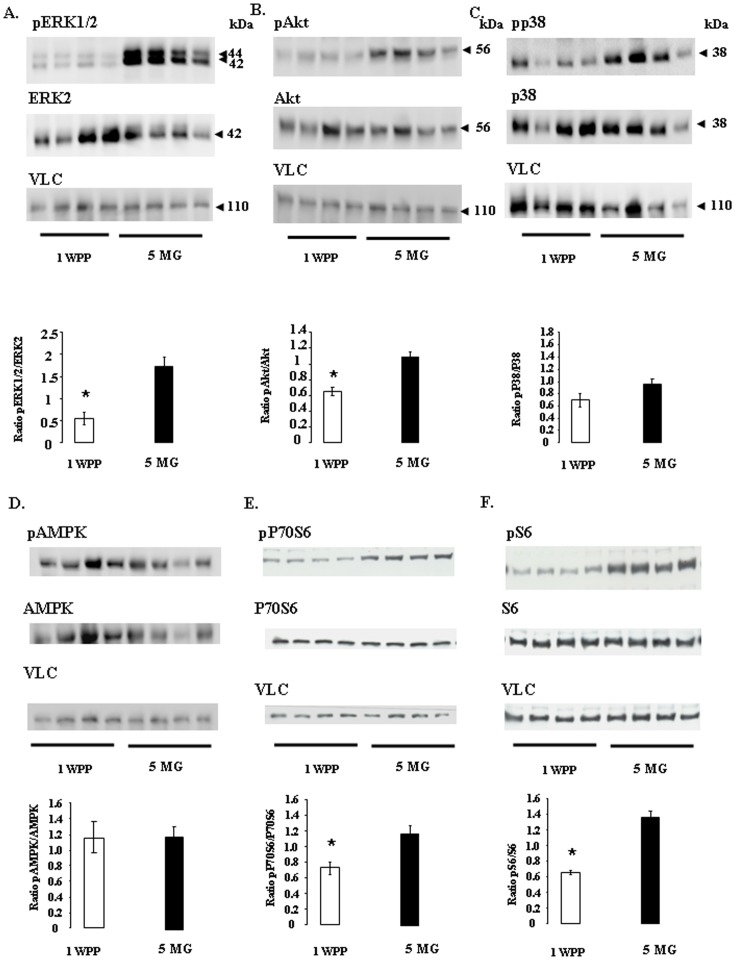
Levels of phosphorylation of MAPK ERK1/2, Akt, MAPK P38, AMPK, p70S6K and S6 kinase in the adipose tissue of dairy cows at one week post partum (1 WPP) and 5 months of gestation (5 MG). Western blots showing the levels of phosphorylation of MAPK ERK1/2 (A), Akt (B), MAPK P38 (C), AMPK (D), P70S6K (E) and S6 (F) in adipose tissue lysates from dairy cows at one week post partum (1 WPP) and 5 months of gestation (5 MG). The levels of phosphorylation of these kinases were normalized by dividing the values obtained for immunoblots of phosphorylated protein by those for immunoblots of the corresponding total protein. Protein levels for MAPK ERK1/2, Akt, MAPK P38, AMPK, P70S6K and S6 were normalized with respect to vinculin levels. The histograms below each gel show the mean ± SEM for two gels, with *n* = 4/group; the groups are 1 WPP and 5 MG. *indicates a significant difference (*p*<0.05).

We then studied various signaling pathways: the MAPK ERK1/2 and P38, Akt, AMPK, P70S6K and S6 pathways (Figure 5). Our results suggest that the level of phosphorylation of MAPK ERK1/2 (Figure 5A), Akt (Figure 5B), P70S6K and S6 (Figure 5E and F) was lower at 1 WPP than at 5 MG, whereas the phosphorylation of MAPK P38 and AMPK (figure 5C and D) did not differ between the two stages. We also found that the levels of Akt, AMPK, MAPKs ERK2 and P38, P70S6K and S6 (normalized with respect to VLC) proteins did not differ between 1 WPP and 5 MG (data not shown).

### Expression of the resistin gene in mature bovine adipocytes

**Figure 6:**
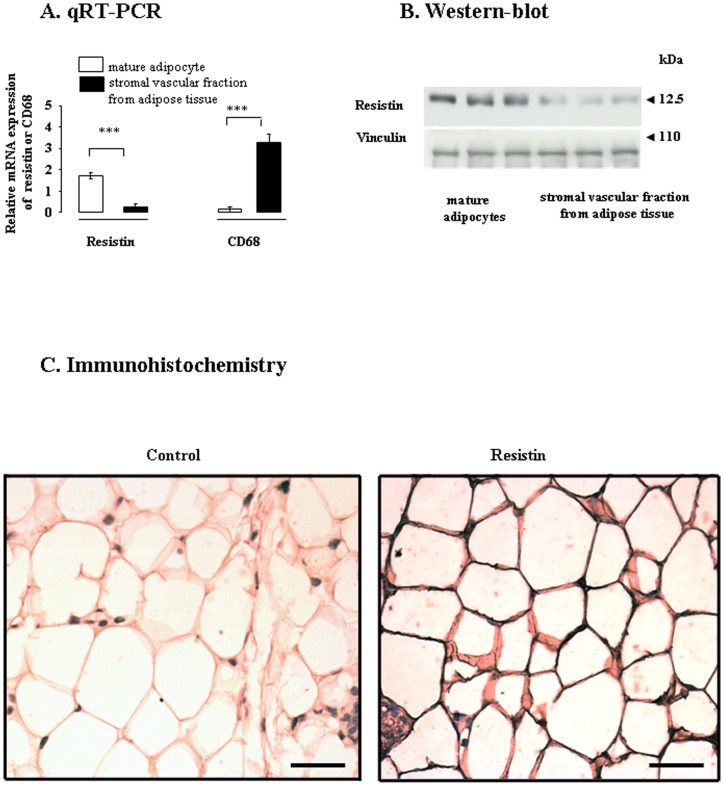
Immunolocalization and levels of resistin protein in bovine adipocytes. Levels of mRNA (A) and protein (B) for resistin in isolated mature bovine adipocytes and in isolated bovine stromal vascular cells, as determined by qRT-PCR and western blotting, respectively. mRNA levels for CD68 (the main cell surface marker of macrophages) was also measured in in isolated mature bovine adipocytes and in isolated bovine stromal vascular cells (A). Relative expression was measured relatively to the geometric mean of 4 reference gene expression [cyclophilin A (PPIA), GAPDH, Actin B (ACTB), and ribosomal protein L19 (RPL19)] by real-time reverse-transcription PCR. Vinculin (VLC) was used as the loading control for immunoblotting. The results are representative of five cell preparations (adipose tissue from one cow was used for one cell preparation). Results are represented as means ± SEM. * indicates a significant difference (*p*<0.05). C. Subcutaneous white adipose tissue from 32-month-old cows was fixed and sectioned, and then subjected to immunochemical analysis to determine of the distribution of resistin. Bar: 100 μm. The image shown is representative of three experiments on three different animals.

We analyzed the levels of resistin mRNA in the subcutaneous adipose tissues of adult dairy cows. As described in the materials and methods, we isolated stromal vascular cells and mature adipocytes and investigated resistin gene expression (by assessing mRNA and protein levels) in both these cell fractions. The quantification of resistin mRNA levels by real-time RT-PCR, indicated that resistin mRNA levels were higher in mature adipocytes than in stromal vascular cells (Figure 6A). Stromal vascular cells include many different types of cell, such as pre-adipocytes, immune cells, fibroblasts, and endothelial cells. In humans, resistin is produced by macrophages rather than adipocytes [7], [8]. We checked that the presence of resistin in the mature bovine adipocytes was not due to immune cell contamination, by also assessing mRNA levels for CD68 (the main cell surface marker of macrophages). CD68 mRNA levels were high in the stromal vascular cells and very low in mature adipocytes (Figure 6A), suggesting that in the resistin was indeed produced by the bovine mature adipocytes. Immunoblotting confirmed that resistin was produced in larger amounts in mature bovine adipocytes than in stromal vascular cells (Figure 6B). We further explored resistin production in bovine adipose tissue, by carrying out an immunohistochemical study with a specific antibody directed against human resistin (the same antibody used for immunoblotting). We observed intense immunostaining for resistin in white adipose tissue (Figure 6C). As a negative control, we replaced the primary antibody with PBS or normal serum. No immunoreaction was observed for the negative controls.

### Effect of recombinant bovine resistin on the release of glycerol from subcutaneous adipose tissue explants and on ATGL (Adipose Triglyceride Lipase) and HSL (Hormone-Sensitive Lipase) mRNA levels in adipose tissue explants

**Figure 7:**
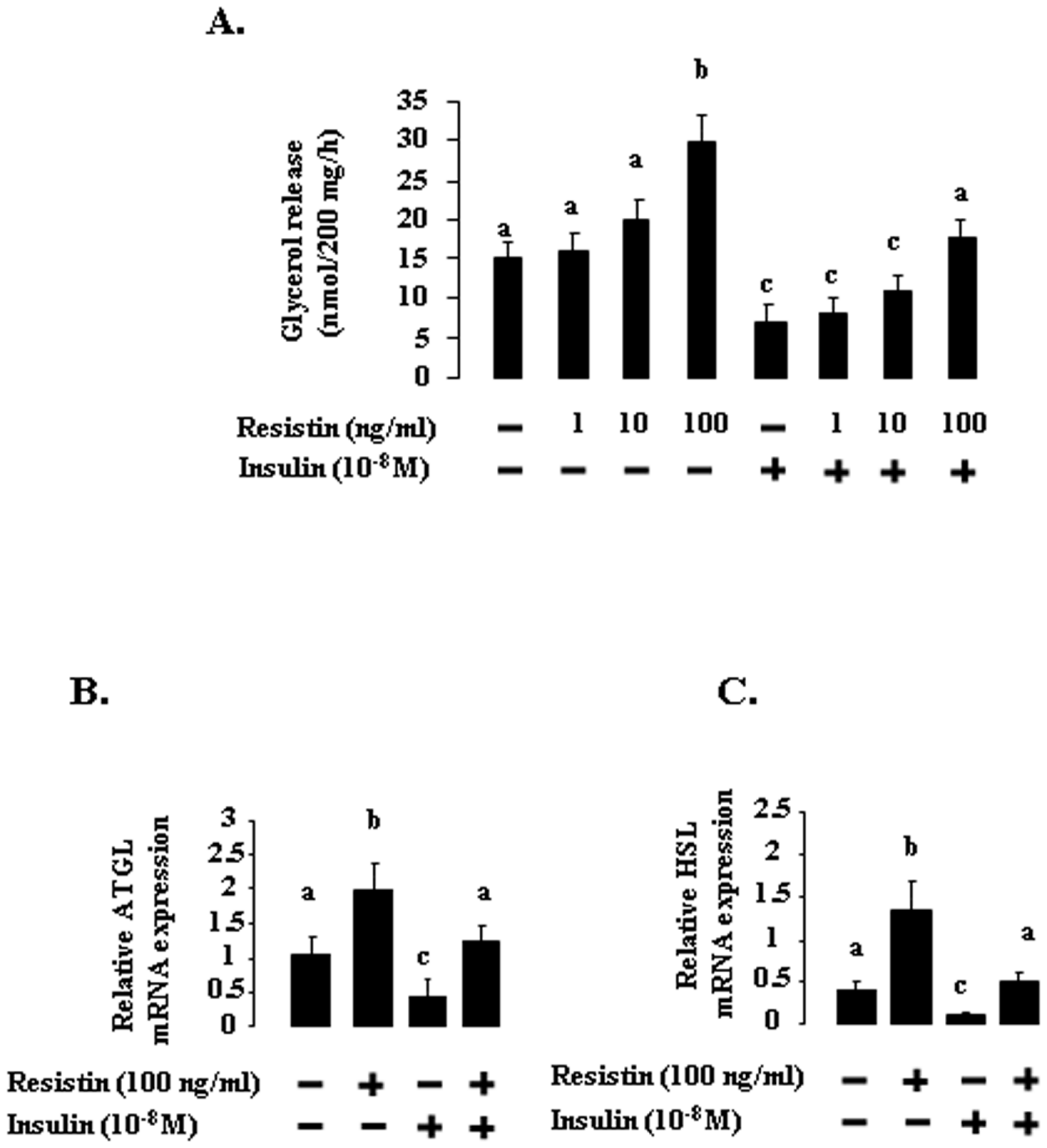
A. Effect of recombinant bovine resistin, in the presence or absence of insulin, on the release of glycerol from subcutaneous adipose tissue explants (*n* = 4 experiments). Data are expressed as nanomoles of glycerol per 200 milligrams of tissue per 1± SEM, and different letters indicate significant differences at the *P*<0.05 level. B. ATGL mRNA and C. HSL mRNA in subcutaneous adipose tissue explants incubated in the presence or absence of recombinant bovine resistin (100 ng/ml) ± insulin (10-8M) (*n* = 4 experiments). Relative expression was measured relatively to the geometric mean of 4 reference gene expression [cyclophilin A (PPIA), GAPDH, Actin B (ACTB), and ribosomal protein L19 (RPL19)] by real-time reverse-transcription PCR. Data are presented as means ± SEM. Different letters indicate significant differences at the *P*<0.05 level.

In the last experiment, we determined the effect of different concentrations of recombinant bovine resistin, in the presence or absence of insulin (10^−8^M), on lipolysis in bovine subcutaneous adipose tissue explants, by measuring glycerol release and levels of mRNA for ATGL (adipose triglyceride lipase) and HSL (hormone-sensitive lipase) after four hours of incubation. Recombinant bovine resistin treatment increased glycerol release in a dose-dependent manner (Figure 7A). Treatment with 100 ng/ml recombinant bovine resistin also abolished the inhibitory effect of insulin on the glycerol release (Figure 7A). As shown in Figure 7B and C, treatment with 100 ng/ml recombinant bovine resistin increased levels of mRNA for ATGL and HSL both in the basal state and in response to insulin (10^−8^ M).

## Discussion

In dairy cows, the onset of lactation increases the total energy requirements due mainly to the nutrient needs of the mammary gland for milk synthesis (Bauman, 2000, Bell, 1995). The hyperphagia required to meet those demands develops slowly, consequently mobilization of endogenous reserves is observed [22], [23]. These metabolic adaptations are coordinated by changes in the plasma concentration of key hormones [22], [23]. For example, the secretion of growth hormone (GH) is elevated in early lactation [22], [24] and promotes the mobilization of nonesterified fatty acids from adipose tissue and their oxidative use by the rest of the body [23]. Cathecolamine-induced lipolysis in adipose tissue (AT) depots is also considered to be the key metabolic pathway for providing endogenous energy in times of high energy demand in the peripartal dairy cow [25], [26]. Recently it has been shown that NEFAs activate the AMPKα signaling pathway to increase lipid oxidation and decrease lipid synthesis in bovine hepatocytes, which in turn, could generates more ATP to relieve the negative energy balance in transition dairy cows [27]. AMPK activation is regulated by various adipokines including adiponectin in bovine hepatocytes [28] and resistin in bovine granulosa cells [29]. Consequently it plays a key role in the control of body fat mass. Here, we show for the first time that plasma resistin concentrations increase one week after calving in a similar manner to NEFA levels in dairy cows. We also found that resistin mRNA and protein levels in adipose tissue were higher one week post partum than at five months of gestation. Conversely, the level of phosphorylation of several components of the insulin receptor signaling pathway in adipose tissue was significantly lower one week after calving than at 5 MG. We also showed that resistin was produced in bovine mature adipocytes and that recombinant bovine resistin increased the release of glycerol and levels of mRNA for ATGL and HSL in adipose tissue explants. Our data suggest that the high levels of resistin in the plasma and adipose tissue observed immediately after calving may contribute to lipid mobilization during early lactation in dairy cows.

Resistin is considered to be a potential factor underlying obesity-mediated insulin resistance and type 2 diabetes. In humans and rodents, serum resistin levels are about 2 to 15 ng/ml [30]–[32], but considerable variability has been noted between species and types of assay. In this study, we obtained values for plasma resistin concentration of 30 to 90 ng/ml in dairy cows. Resistin is produced principally by adipocytes in mice, whereas it is produced predominantly by peripheral blood mononuclear cells, macrophages and bone marrow cells in humans [7], [8]. The production of resistin in bovine adipose tissue has already been reported, but the cell type responsible for this production was not identified [15]. We detected resistin in mature bovine adipocytes.

We also demonstrated that plasma resistin concentration was significantly higher one week after calving than before calving or six weeks postpartum. Consistent with these results, we found that resistin mRNA and protein levels in subcutaneous adipose tissue were higher at 1 WPP than at 5 MG, suggesting that the high plasma concentrations of resistin at 1 WPP are generated by the adipose tissue. Lactation in dairy cows is known to be associated with many metabolic changes, including the loss of a large amount of adipose tissue. In our study, the animals lost more than 1 kg of body weight/day during early lactation. These results are consistent with those of Jarrige (1989) that indicates a mobilization of body fat from 15 to 60 kg after parturition [33]. As expected, plasma NEFA concentration was also found to have increased considerably at 1 WPP, reflecting a high level of lipid mobilization [34]. The plasma concentration profiles of NEFAs and resistin were similar during the peri-partum period. However, the nadir for plasma resistin was reached at 4 WPP while those for plasma NEFAs at 6 WPP.

*In vitro*, in bovine adipose tissue explants from animals at about the same physiological status (between one and two months after calving), we showed that recombinant bovine resistin at a concentration of 100 ng/ml increased the release of glycerol and the expression of the ATGL and HSL genes. This concentration is physiologically relevant because we measured a plasma resistin concentration of about 90 ng/ml at one week post-partum when plasma NEFAs were high. In adipose tissue of cows, hydrolysis of triacylglycerols is mediated by hormone-sensitive lipase (HSL) under stimulation of catecholamines [35]. Concomitantly with the decrease in HSL expression, plasma NEFA levels are high during the early postpartum period [35]. Resistin induces lipolysis in human adipocytes [36]. The secretion of GH is also high in early lactation. Growth hormone stimulates the mobilization of NEFAs from adipose tissue by inhibiting insulin-mediated lipogenesis and increasing the lipolytic response to beta adrenergic signals [37]. However, the regulation of GH receptor expression in the adipose tissue of early lactation dairy cows is unclear [38], [39]. In rodents or human, GH increases resistin gene expression in white adipose tissue or serum resistin levels [40], [41]. Thus, we can hypothesis that resistin could participate to the in vivo GH effects on the adipose tissue of dairy cows. However, we observed that resistin induces in vitro mRNA expression of ATGL and HSL mRN on adipose tissue explants suggesting that resistin could also act independently of GH.

We found that plasma insulin and glucose concentrations followed patterns typical of the peri-partum period, declining sharply at 1 WPP [2]. By contrast, plasma resistin levels and the levels of resistin mRNA and protein in adipose tissue increased during this period. Komatsu *et al*. also reported higher levels of resistin production in adipose tissue and lower plasma insulin concentrations in dairy cows at peak lactation (around 8 WPP – 10 WPP) than in dry animals [15]. Plasma concentrations of two other adipokines, leptin (the most studied) and adiponectin, have been analyzed in dairy cows. Leptin regulates food intake, energy partitioning and adipose tissue deposition during both short- and long-term changes in nutritional state [12]. In dairy cows, plasma leptin concentrations are high before calving, proportionally to body condition score (BCS); they then decrease at calving and then remain low even when energy status improves [12], [42]. In our study we observed a significant negative correlation between plasma resistin levels and energy balance and plasma resistin levels and dry matter between WPP1 and WPP2.After calving, hypoleptinemia may contribute to peripheral insulin resistance [12]. Indeed, unlike resistin, leptin is known to increase insulin sensitivity [43]. Plasma adiponectin concentrations have recently been investigated in dairy cows. Adiponectin, like leptin, increases insulin sensitivity in various species. Mielenz *et al*. (2013) showed, by western blotting and ELISA, that, in multiparous Holstein-Frisian dairy cows, plasma adiponectin concentration decreased from day −21 antepartum, reaching a trough at day 1, and increasing thereafter, with the highest values attained on day 14 postpartum [44]. Giesy *et al*. (2012) also obtained similar results [45]. The profile of adiponectin protein levels in adipose tissue reported here are at odds with the variations of plasma adiponectin concentration previously described by [44]–[47]. Indeed, our results show that adipose levels of adiponectin protein are lower at 1 WPP than at 5 MG. Koltes and Spurlock (2012) and very recently Saremi *et al.* (2014) observed a decrease of the adiponectin mRNA in subcutaneaous adipose tissue throughout the transition period [48], [49]. Moreover, Lemor *et al*., 2009 showed that plasma leptin concentrations and the levels of two adiponectin receptors (AdipoR1 and AdipoR2) in subcutaneous adipose tissue were lower one week before calving than three weeks post partum [13]. It is well known that at the beginning of lactation plasma insulin levels are decreased compared to the pre partum level [50] because of reduction of pancreatic function [51], and insulin response to glucose infusion is reduced [52]. However, we can also hypothesize that an increase in plasma resistin levels and a decrease in plasma leptin and adiponectin levels towards lactation may contribute to the decrease in insulin sensitivity. However, further experiments are necessary to demonstrate this hypothesis.

As pointed out above, the molecular mechanism underlying the decrease in insulin sensitivity in peripheral tissues (adipocytes and muscles) during early lactation in dairy cows is not yet well understood. However, it is well established that bovine adipose tissue adapts pre-partum with a shift towards NEFA mobilization rather triglyceride accumulation [53]. Using tail-head subcutaneous fat, Sadri *et al*. (2010) showed a decrease in the abundance of mRNA for GLUT4 and GLUT1 on day 1 post partum, potentially reflecting a physiological adaptation of the adipose tissue [4]. However, they observed no change in gene expression for IRS-1, IR and P85 or P110 (regulatory and catalytic subunits of PI3K). Our findings confirm these results at the protein level for IR, IRS-1, IRS-2, Akt, MAPK ERK1/2 and P38, AMPK, P70SK, S6K and IGF-1R, at 1 WPP and 5 MG. We also observed a significant decrease in the tyrosine phosphorylation of IR, IRS-1, IRS-2, P70S6K, S6, Akt and MAPK ERK1/2 that can be explained by the strong decrease in the plasma insulin levels one week after calving. However, IGF-1Rbeta, P38 MAPK and AMPK displayed similar levels of phosphorylation at both these stages. The insulin receptor (IR) and the insulin-like growth factor-1 receptor (IGF-1R) belong to the same subfamily of receptor tyrosine kinases [3], [54]. In our study we showed that plasma insulin concentrations are low one week postpartum and increase after. We did not measure IGF-1 plasma concentrations but it is well known that plasma IGF-I concentrations are also low during the week following parturition [55]. Here, we observed that IR beta subunit tyrosine phosphorylation was decreased WPP1 as compared to 5MG whereas tyrosine phosphorylation of IGF-1R was similar in both physiological states. These data suggest that even if IR and IGF-1R are two tyrosine kinases receptors very closed they are differently regulated in bovine adipose tissue. However, the physiological meaning of this different activation between IR and IGF-1R remains to be determined. The p38 MAPK signalling pathway is not specific to insulin or IGF-1. It allows cells to interpret various external signals and respond by generating a plethora of different biological effects. This can explain why P38 MAPK phosphorylation was unchanged between WPP1 and 5MG. AMPK plays an important role in the regulation of energy metabolism. Previous studies have reported higher levels of AMPK phosphorylation in bovine adipose tissue on day 1 postpartum than on day 21 prepartum [56], [57]. Based on these results, antilipolytic effects of AMPK for dairy cows have been addressed [56]. However, consistent with our results, Locher et al (2012) reported no variation of AMPK phosphorylation between days 1 and 21 postpartum and according to the degree of NEB. They suggested that AMPK activation is dependent on the lipolysis. In our study even if plasma NEFAs are significantly different between WPP1 and 5MG we observed no change in the AMPKα phosphorylation. However, in our study we determined phosphorylation of both AMPKα isoforms (α1 and α2) (not only AMPKα1 as in [56]) and the location of subcutaneaous adipose tissue was also different (region of the tailhead for [56] vs neck for our study).

## Conclusion

In our study we have showed that resistin is present *in vivo* in mature bovine adipocytes. We also observed that plasma resistin levels were high one week after calving and positively correlated with plasma NEFA levels and negatively with milk yield, dry matter intake and energy balance between WPP1 to WPP22. Finally, we have shown that recombinant bovine resistin increased the mobilization of lipids *in vitro* in adipose explants. Further studies are necessary to determine if resistin is *in vivo* involved in the adipose tissue mobilization during early lactation.

## Supporting Information

Figure S1 **Resistin protein production in mature bovine adipocytes, with recombinant bovine resistin used as the control.**
(TIF)

Table S1 **Value of the RNA integrity number (RIN) for each RNA sample used in the study.**
(DOC)
