# Supplementary material for: Resistin in Dairy Cows: Plasma Concentrations during Early Lactation, Expression and Potential Role in Adipose Tissue
Source: PLoS One. 2014 Mar 27;9(3):e93198. doi: 10.1371/journal.pone.0093198 (PMC3968062; doi:10.1371/journal.pone.0093198)
Supplement: Table S1 — Value of the RNA integrity number (RIN) for each RNA sample used in the study. (DOC) [file pone.0093198.s002.doc]

**Supplemental table 1 : Value of the RNA integrity number (RIN) for each RNA sample used in the study**

**A. Value of the RIN for each RNA sample used in figure 3**

| **Cow number** | **Adipose Tissue at WPP1** | **Adipose Tissue at 5 MG** |
| --- | --- | --- |
| **1** | **7.9** | **8.1** |
| **2** | **8.1** | **8.3** |
| **3** | **8.2** | **7.9** |
| **4** | **8.5** | **8.2** |
| **5** | **8.1** | **7.7** |
| **6** | **7.9** | **7.9** |
| **7** | **8.1** | **8.0** |
| **8** | **8.2** | **8.2** |

**B. Value of the RIN for each RNA sample used in figure 6A**

| **Sample** | **Isolated bovine mature adipocytes** | **Stromal vascular fraction**  **from adipose tissue** |
| --- | --- | --- |
| **1** | **8.1** | **7.8** |
| **2** | **8.2** | **8.5** |
| **3** | **8.1** | **8.6** |
| **4** | **8.1** | **8.9** |
| **5** | **8.0** | **8.1** |

**C. Value of the RIN for each RNA sample used in figure 7B and C**

| **Sample** | **Control** | **Resistin** | **Insulin** | **Resistin +Insulin** |
| --- | --- | --- | --- | --- |
| **1** | **8.1** | **8.2** | **8.3** | **7.9** |
| **2** | **8.3** | **7.8** | **8.0** | **8.1** |
| **3** | **8.2** | **8.1** | **7.9** | **8.2** |
| **4** | **7.9** | **8.2** | **8.4** | **8.5** |
